# Study of Uprooting in Pediatric Cancer Care for Children From the French West Indies and Guiana Treated in Mainland France: A Qualitative Study

**DOI:** 10.1002/pon.70242

**Published:** 2025-07-29

**Authors:** Chloe Vanlaer, Claire Dichamp, Narcisse Elenga, Frederique Delion, Graziella Raimondo, Sylvie Fasola, Sandrine Haghiri, Katell Michaux, Yves Hatchuel, Benjamin Faivre, Etienne Seigneur, Marie Simbozel, Quentin Neven, Céline Khouri, Arthur Felix, Anais Ogrizek

**Affiliations:** ^1^ Department of Pediatric Oncology Institut d'Hématologie et d'Oncologie Pédiatrique Hospitalisation à domicile Lyon France; ^2^ Department of Pediatrics Children Hematology and Oncology Unit University Hospital of Martinique Martinique France; ^3^ Department of Pediatrics Cayenne Hospital Cayenne France; ^4^ Department of Pediatrics Guadeloupe University Hospital Pointe‐À‐Pitre France; ^5^ Department of Pediatrics Margency Croix‐Rouge Hospital Margency France; ^6^ Department of Pediatrics Medical Clinic Edouard Rist Paris France; ^7^ Children Hematology and Oncology Unit Pediatric and Reeducation Hospital Bullion France; ^8^ Interdisciplinary department of supportive Care and psychooncology Curie Institute Paris France; ^9^ Department of Child and Adolescents Oncology Gustave Roussy Institute Paris‐Saclay University Villejuif France; ^10^ Department of Pediatric Hematology Robert Debré Hospital Paris France; ^11^ Department of Pediatric Hematology‐Oncology Trousseau Hospital Paris France; ^12^ EpiCliV Research Unit University of the French West Indies Martinique University Hospital Fort‐de France France; ^13^ Department of Adult and Child Psychiatry Martinique University Hospital Fort‐de‐France France

**Keywords:** Afro‐Caribbean, care pathways, children oncology, pediatric, uprooting

## Abstract

**Background:**

A diagnosis of childhood cancer has a profound impact on families, especially when treatment requires relocation. Children from the French West Indies (FWI) and French Guiana (FG) are often treated in specialized centers in mainland France, resulting in physical and cultural uprooting that poses unique psychosocial challenges.

**Aims:**

This study explores the impact of uprooting on the dynamics and well‐being of these families.

**Methods:**

This qualitative observational study used semi‐structured interviews with parents of pediatric cancer patients from the FWI and FG. Eligible participants were French‐speaking primary caregivers with parental authority who accompanied their children for treatment in mainland France. Interviews were conducted between November 2023 and February 2024, recorded, transcribed, and analyzed using Interpretative Phenomenological Analysis with NVivo software.

**Results:**

Among the 10 families interviewed, uprooting significantly disrupted family dynamics and required complex adjustments. Parents reported challenges in accessing resources to manage the stress of their child's diagnosis and treatment, but expressed overall satisfaction with the healthcare received. Psychological support was essential, with many finding comfort in religious practices. Financial and social difficulties were common. Children generally adapted better than expected but struggled with separation from their familiar environment, while siblings often felt abandoned.

**Conclusion:**

This study is the first to examine the impact of uprooting on childhood cancer treatment for families from this region. Providing care closer to home may reduce psychological and financial burdens. Recommendations include establishing regional pediatric oncology units and increased support from social services. Future research should focus on non‐accompanying parents, siblings, and schooling to understand the long‐term effects on survivors and develop targeted psychological and social interventions.

**Abbreviations**
EVASAN: Medical Evacuation
FG: French Guiana
FWI: French West Indies
INSEE: French National Institute for Statistics and Economic Studies
IPA: Interpretative Phenomenological Analysis
NA: Non‐Applicable
UCNT: Undifferentiated Carcinoma of Nasopharyngeal Type

## 1 Introduction

The diagnosis of cancer is a major upheaval for children and their families [1]. Studies have shown that long‐term monitoring of these patients reveals a considerable psychological effect mainly symptoms of posttraumatic stress disorder (PTSD), with functional impairment and/or clinical distress [2]. Additionally, the educational and social prospects of children who survive cancer are often negatively impacted with a higher risk at adulthood of lower educational levels, unemployment, annual incomes below $20,000 and celibacy [2]. The French West Indies (FWI) and French Guiana (FG) have a combined population of approximately 300.000 children under 18 years of age [3]. There are 2 university hospitals and regional competence centers in these regions, approved by the French Ministry of Health. The healthcare system in the French overseas departments mirrors that of mainland France, with accessible and reimbursed care.

In France, pediatric oncology care is organized within a national network of reference centers that aim to provide every child with the highest quality treatment, as close to their home as possible, while ensuring access to standard or advanced therapies when necessary. During the study period, the healthcare pathway for a child diagnosed with cancer in FWI or FG typically involved several steps. The initial or suspicious diagnosis was made at local center in FWI and FG, and departure for mainland France was arranged promptly. Patients were then referred to pediatric oncology reference centers in mainland France, mostly in Paris area due to interregional agreements with world‐renowned pediatric oncology hospitals, to undergo intensive treatment. Upon completion of this phase, the children returned to their local centers in FWI and FG for maintenance treatment and/or follow‐up. The need for FWI and FG patients to be treated at reference centers in mainland France often requires them to travel long distances and live away from home, typically for 4–24 months, in addition to being diagnosed with a serious illness. A recent quantitative study of health evacuations for pediatric cancer in La Reunion island, involving 105 children between 2015 and 2019, reported significant familial, psychological, financial and social challenges [4].

The aim of our study was to explore the uprooting experience of the children and their family through semi‐structured interviews with the main accompanying parent. These interviews addressed various personal, family, socio‐cultural, educational and psychological aspects. The child's experience of readjustment upon returning home after a long stay in mainland France for treatment was also examined.

## 2 Methods

### 2.1 Study Design

This phenomenological study is based on the analysis of semi‐structured interviews with parents living in the FWI or FG whose child was treated for childhood cancer in mainland France.

### 2.2 Inclusion and Exclusion Criteria

The inclusion criteria were as follows: parents accompanying their child with pediatric cancer (< 18 years) including hematologic malignancy or solid tumor, regardless of the treatment received, diagnosed within the last 5 years, who were resident of the FWI or FG at the time of diagnosis. Participating parent had to be French‐speaking and provide prior written consent to participate in the study. Parents were included if the children had received most of their cancer treatment in mainland France and stayed there for at least 4 consecutive months. Parents were excluded if they were not the main caregiver of their child, if their child had died or was in palliative care, or if they did not have parental authority. Parents who were experiencing strong emotions that may be relevant for an acuteization of an undisclosed psychiatric disease (post‐traumatic stress disorder, anxiety disorder, depression) were excluded as a precaution; this point was assessed by the principal investigator, who is a physician.

### 2.3 Data Collection

Recruitment was based on medical data provided by the local hospitals of the FWI and FG (University hospital of Martinique, University hospital of Guadeloupe, Cayenne hospital, FG) and by the reference centers in mainland France (Robert Debré university hospital, Armand Trousseau university hospital, Gustave Roussy Institute and Curie Institute). The 14 families were selected based on the underlying pathology to ensure a diverse panel of tumors, the recency of treatment to avoid memory bias, and the remission status of the child.

All eligible parents were contacted by phone by the principal investigator, who provided oral information about the study. An information sheet about the study was emailed to them, and if the parent gave consent and had met no exclusion criteria, an interview was scheduled.

An interview guide was drawn up with open‐ended questions drafted by the research team (Supporting Information S1), based on a review of the literature that helped identify key aspects of the subject: the experience of leaving [5, 6], family organization [7, 8], the behavior and experience of the long‐distance stay and isolation for care [9, 10], the impact on the family [11, 12, 13], the return and post‐cancer experience [14]. The interviews were conducted by the principal investigator with one or both accompanying parents, via video conference or face‐to‐face at the hospital in Martinique.

The research group discussed and revised the questions after each interview, incorporating additional themes raised by the parents as they emerged. During each interview, open‐ended questions were used to initiate discussion of the research themes. At the end of each interview, participants were systematically asked if they wished to discuss any other issues or if they had any suggestions for improving the organization of care and the management of childhood cancer in this context.

### 2.4 Data Analysis

The analysis was guided by a phenomenological approach, specifically using Interpretative Phenomenological Analysis (IPA) to explore the meaning that participants construct from their lived experience. IPA was chosen because phenomenology is considered the most appropriate theoretical framework for exploring the complex, ambiguous and emotionally charged experiences of a small group [15]. This method aims to identify the meanings that individuals attach to their experiences, using their own detailed accounts and in their own words, with each case examined independently [15]. To ensure the validity of the findings through triangulation, each interview was analyzed by at least two different researchers. The analysis was carried out using NVIVO 14 software, which was used to identify themes and meta‐themes.

The analysis process involved several steps: interviews were recorded, transcribed, anonymized, listened to and reread by the principal investigator, read and reread several times, with transcripts annotated at each reading. The annotations were then grouped into themes reflecting the main ideas identified by the researchers. Links between themes were identified and developed to create a coherent and logical organization. Meta‐connections between themes were then identified and themes were grouped into broader axes describing global aspects of participants' experiences. Each axis contained several themes, which in turn consisted of the researchers' annotations on the interview transcript. At each stage of the analysis, the researchers ensured the coherence of the links established between different themes by constantly cross‐referencing between interviews. If a new theme emerged during the analysis of an interview, previously analyzed interviews were revisited to look for elements that might correspond to this new theme.

### 2.5 Ethics

The study was approved by the Institutional Review Board of the Martinique university hospital under number 2022/227.

## 3 Results

**FIGURE 1:**
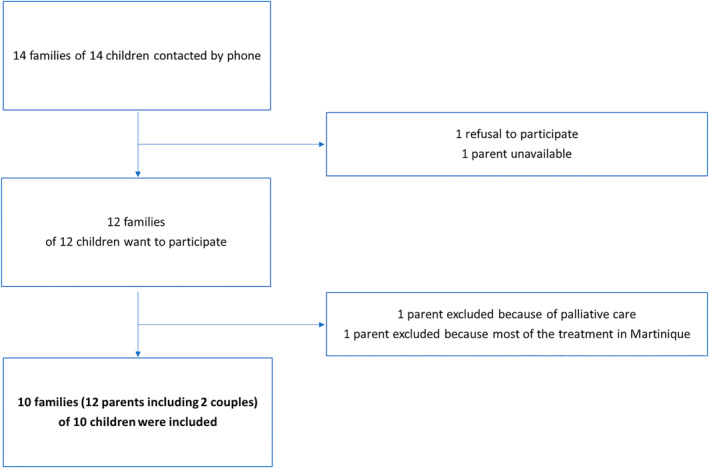
Flowchart.

The flowchart of the study is shown in Figure 1. Parents of 14 children were contacted and we included a total of 12 parents from 10 families. There were 12 participants: 7 mothers, 1 father and 2 couples (both father and mother). This resulted in a total of 10 semi‐structured interviews (2 interviews with both mother and father) conducted between November 2023 and February 2024. These interviews were conducted either in Martinique at the University Hospital or by videoconference and were subsequently analyzed. The interviews lasted between 41 and 90 min, with a median of 62 min. Table 1 shows details of the demographic characteristics of the parents and patients, the children's cancer subtypes and the length of stay in mainland France. Of the parents interviewed, 3 were single mothers. Two mothers were unemployed at the time of diagnosis; the other parents were employed. Half of the parents were homeowners and the other half were tenants.

**TABLE 1:** Parents and patients characteristics at diagnosis.

Patients	Pathologies according to international classification of childhood cancer (ICCC)	Duration of stay in mainland France for care (< 6 months/6–12 months/> 12 months)	Main caregiver parent(s)	Parent(s) interviewed	Working parents	Housing	Place of residence	Age (< 5 years – 5–15 years ‐ > 15 years)	Siblings	Schooling (Yes/no)
A	Lymphoma	< 6	Father	Father	Mother and father	Owner	Martinique	> 15	1 brother 1 sister	yes
B	Cerebral tumor	< 6	Mother	Mother	Mother and father	Owner	Martinique	5–15	1 sister	yes
C	Lymphoma	6–12	Mother	Mother	Mother and father	Owner	Martinique	5–15	0	yes
D	Kidney tumor	6–12	Mother and father	Mother and father	Mother and father	Owner	Guadeloupe archipel	< 5	1 sister	yes
E	Sympathetic nervous system tumor	< 6	Mother and father	Mother and father	Father	Renter	Martinique	< 5	1 half brother 1 half sister	NA
F	Kidney tumor	6–12	Mother	Mother	Father	Renter	Martinique	< 5	0	NA
G	Sarcoma	< 12	Mother	Mother	Mother and father	Renter	Martinique	5–15	1 sister	yes
H	Carcinoma	6–12	Mother	Mother	Mother and father	Renter	Guadeloupe	5–15	1 sister 1 brother	yes
I	Lymphoma	6–12	Mother	Mother	Mother	Renter	French Guiana	5–15	1 brother	yes
J	Sarcoma	< 6	Mother	Mother	Mother and father	Owner	Martinique	5–15	0	yes

Abbreviation: NA: Not applicable.

The majority of the children (*n* = 7, 70%) lived in Martinique, the others in FG, Guadeloupe and its archipelago. Among the 10 children whose parents were interviewed, the sex ratio was 1:1. The median age at onset was 6 years (range: 0.5–17 years). Three children were singletons and 7 had siblings. The median length of stay in mainland France for treatment was 6 months (range: 4–12 months). All of the parents were interviewed within 2 years of the end of their child's treatment. Notably, two families chose to move permanently to mainland France for personal reasons and also for the reassurance of being close to a reference center for their children's disease. Four main themes were identified: (i) The role of the parent: focus on the child and management of complex logistics, (ii) the interdependence of the parent‐child pair with the environment, (iii) Remaining a child above all else: during illness and in long‐distance care, and (iv) a positive perception of partial care in Martinique. Please refer to Table 2 for quotes.

**TABLE 2:** Superordinate themes, themes and quotes.

The parent's role: focusing on the child while managing complex logistic	**Hasty and urgent departure induces lack of organisation**	
	Quote 1	“We didn't expect this at all. A. was in the middle of a competition, and it came as a crushing blow. We really, really weren't expecting it. So, how were we organized?” We weren't organized at all!” Father A
	Quote 2	“It was quite a shock to leave everything. In 1 week, to leave everything without really knowing where we were going”. Mother B
	Quote 3	“I don't even know if we can talk about organization, because for me, it was anarchy. We were told we were leaving… on Saturday. On Saturday, we were told that on Thursday. All the while knowing that we'd planned to go home. We didn't think our son had anything in particular”. Mother H
	Quote 4	“We were told, ‘Your child is sick’. And then we were told, ‘here, we're going to put you on this plane here and you're going to leave’”. Mother H
	**The sick child: a new priority**	
	Quote 5	“Nothing was organized, my work… I didn't give a damn. From one day to the next, he [his son] had to leave. That was my priority. There was nothing more important, so we got organized. […] My brain had really gone into warrior mode. It's like, we've got to save my son, it doesn't matter what's going on next door, it doesn't affect me or anything.” Father A
	Quote 6	“I didn't think about it, she didn't have any time to lose […] In fact, my objective was my daughter, that she be cured in the first place, since that was the first objective. You don't have a choice; you have to do it. When you're determined to do something, you have to go all out. That's the way it is. ” Father E
	**The Potomitan parents**	
	Quote 7	“I had my crying spells afterward, because during C.'s treatment I never actually cried. I don't know, I was really focused on him, so there was no time to break down.” Mother C.
	Quote 8	“It was already complicated for I., but if on top of that I'm not up to it, it's not worth it!” Mother I
	Quote 9	“Every time, as a parent, I was divided between wanting to be tougher on him and sometimes wanting to be more lenient. It's not easy to find the happy medium under these conditions.” Mother G
	**Lost in translation**	
	Quote 10	“And then, behind, we tried to manage, to look for where we were going to sleep. So, we went to the hotel we'd been told about. They told us ‘No, we have no room, we have nothing’. I said, ‘well, listen, we're going to pay out of our own pocket because we don't know where to sleep’. […] That's it, we put it all in. We put everything in. That's it.” Father A
	Quote 11	“I took hotel nights because I'm with the little brother, […] if I leave him at 3 years old, he won't understand. […] We left in a hurry; he knew there was a health problem. So already, all this is very distressing. […] If I hadn't had a nest egg, I don't know how we'd have managed. Mutual insurance company reimbursements are next to nothing. You have to pay everything up front. It's horrible. It's pathetic.” Mother I
	Quote 12	“So, the hospital isn't doing its job. The hospital is there to treat. It's not there to find housing for families. But what I find… what I find crazy is that… we're French, we're overseas, we've made the choice to be cared for properly. And for that, we have to manage. And we have to pay for everything. It's a horror…” Mother I
	Quote 13	“What really bothers me is that, in the long term, you can't continue to receive your full salary from your employer. […] I don't see how it was illegitimate for me to receive my salary. And that's not normal. I think it needs to be reviewed.” Mother G
	**Struggling through adversity**	
	Quote 14	“I'm on my own. […] I had to spend every other night with my daughter. I couldn't accompany my daughter as I wanted to. Because there was the little brother on the other side, because nothing was done so that we could stay together or close by.” Mother I
	Quote 15	“He [the father] had to go to work in the cold. We're not used to all that.” Mother F
	Quote 16	“I told myself I wasn't going to leave our daughter like that. Purchase. Store right away. She was dressed. Coat, down jacket, socks, tights, boots, everything. Our daughter had to be warm because it's cold there.” Father E
The interdependence of the parent‐child pair with the environment	**Children and parents standing together**	
	Quote 17	“And then, between treatments, as soon as I. was more or less fit, we'd get moving. We were going to discover lots of things. We went to Brittany, we went to Belgium, we went to the mountains, and then afterward, we moved around a lot in Paris: museums, shows. It was all about clearing our heads and having fun. We spent the first of January at the Vincennes zoo, walking around with our sandwiches.” Mother I
	Quote 18	“So that's what was important for us, to have the right to become a family again, because we were fine together. Together was fine, so for me, we absolutely had to be together, whatever the cost.” Mother H
	Quote 19	“I work in the civil service and he left his job because he [the father] didn't want the family to be separated, so we left as a family.” Mother B
	Quote 20	“We'd already made arrangements for the two of us to leave, because it would be much easier for us. […] At one point my wife went back to Martinique and when she came back, it was much harder when she wasn't there. It was much more difficult because I know she was managing the logistics from Martinique. I know it wasn't easy. […] Right, so it wasn't easy for me because my wife wasn't there, my children, my other children weren't there. So, it was difficult. […] And at that point, we repatriated everyone to France. And that was much easier. It really was a lot easier.” Father A
	**A shared experience**	
	Quote 21	“On the contrary, it brought us closer together, it welded us together more, it was able to unite the family around A., around everything.” Father A
	Quote 22	“Frankly, even if I think it was bad, even if I think an ordeal like this can only strengthen bonds. We're both going through it, even if we were not necessarily on good terms or anything. It's both our children, so it hurts in the same way to know that your child has such a horrible disease. So, we have no choice but to support each other, really.” Mother F
	Quote 23	“It's been a relief. It was a relief, yes. […] My daughter's illness has enriched us all. It's a strange thing to say, but we've gained a lot from what we've been through. It wasn't easy, far from it. But we've all grown from it, in one way or another. And I wouldn't do anything differently. Well, maybe some things to make it less scary. But basically, no. It's almost thanks to cancer. It's almost that.” Mother I
	**Intricacies of family reorganization**	
	Quote 24	“So, I had the feeling that she [G's sister] hadn't lived through this period very well, since she already felt a bit more tossed about. […] There, I felt that it did indeed bother her a little, that it disturbed her, but well, I didn't have much choice.” Mother G
	Quote 25	“It's complicated for her [H's sister], but it's complicated for me because I can't, I can't yet restore the balance we had in our family. She doesn't take it well, I feel she doesn't take it well. Even today.” Mother H
	Quote 26	“Afterward, it's not easy for the couple, unfortunately. It's not easy at all, because one of us stayed, and the other went back home and resumed his little life. Whereas ours is a little bit stopped in time with the pathology”. Mother C
	**The other side of the coin**	
	Quote 27	“When we return to reality, we have very little support. We have the impression that we're being told, 'You're cured, what's the problem? Get on with your life’. […] Honestly, it's really… Because there's the during, there's the before, but above all there's the after. After, it's supposed to be the part where you're cured, so ultimately, there's no point.” Mother C
	Quote 28	“Today, the question of going back [to the West Indies] hardly arises at all, in fact. I want to go home, that's true. I can't adapt here [in mainland France]. I'll never make it. […] I think I need to see a shrink because I can't live here. […] So for the moment, I just can't get used to it.” Mother H
	Quote 29	“And I really had the impression that I was left to my own devices, that I had no psychological follow‐up, because it doesn't stop there, our child was sick, but it doesn't stop at that point. And that's really the impression I've had up until now, that I've been abandoned. So, the slightest little thing sends me into a panic. I'm stressed every time something happens. I try to… detect why he's tired today, ‘is there a relapse?’ […] so really I'm in a permanent state of daily stress.” Mother H
	Quote 30	“So here again we say to ourselves, if there's a recurrence, if there are other health problems, wouldn't it be better to go back? [to mainland France] and already be there so that it's not as complicated as it was the first time.” Mother G
	**Family's support network**	
	Quote 31	“So, we did it fast, fast, fast, fast. We made arrangements with my parents, who were still on the island at the time, because they're not on the island all the time, so that they could look after the older sister.” Mother D
	Quote 32	“It's true that when you live through a moment like that [diagnostic of child cancer], at least when you're at home, you can go cry to Mom. [She laughs] We can go and give Mum a little hug, which will reassure us.” Mother F
	Quote 33	“As there are many children who live far, far away, who are foreigners [in Mcdonalds parents' house]. So, they [associations] make sure they can organize activities for the children as well as for the parents. So, whether it's tea parties, outings, all that, they're associations that organize it for the children as well as the parents, so that their daily lives become less burdensome as the treatment progresses.” Father E
	Quote 34	“So, we supported each other well there [at the parents' house] too. Knowing that we're parents and that we all have one thing in common, which is that we have a sick child. So, all that also gave us a bond at the [reference center]. There are some people we really enjoyed talking to, in fact a little bit of everything.” Father E
	Quote 35	“When we first came to [reference center], what was nice was that there was a little church, but it was really close by. I used to go there quite often, very very often. I asked for masses for my son. So, I made donations for my son, and for the children who are sick too.” Mother J
	Quote 36	“On the other hand, we were also extremely lucky in terms of the logistical infrastructure, if you can call it that, family and friends. Because it's true that we had an apartment at our disposal, which means we didn't have to pay a month's rent in the center of Paris. I mean, that's impossible for most people. So, we had superb conditions.” Parents D
	**The hospital: The lighthouse in the storm**	
	Quote 37	“We ask ourselves all these questions: 'Will the treatment work?’ Afterward, we were reassured that it was working at least 97 or 98%, so we said to ourselves that it should work, because when we arrived, we didn't know anything about it. I just told myself that I had to be there.” Mother C
	Quote 38	“When I saw that it was the [reference center], it's one of the best hospitals in Europe and everything. Frankly […] it gave me confidence in any case.” Father A
	Quote 39	“It's life outside the [reference center] that we need to help. Because it's not just that. You're not in hospital for 6 months. So, it's all about organization, logistics and support” Mother I
Remaining a child above all else: in illness and in long‐distance care	**Child's acceptance of care**	
	Quote 40	“But at first, he didn't really want to leave. […] So I was really asking him to be more cooperative with the medical protocol. And sometimes he refused medication, which wasn't possible because I told him that the more he refused, the longer we would stay in mainland France.” Mother G
	Quote 41	“He didn't want his brothers to see him like this, even if we saw each other by videotape, but he didn't want us to see him sick.” Father A
	**Child's need for support**	
	Quote 42	“I think it was also important in the end that all 4 of us were able to go away, because even though there were a lot of changes, the family unit was still there, and I think that helped B. a lot”. Mother B
	Quote 43	“Because, in fact, G. really likes his classmates. He really likes going to school, especially to socialize, not necessarily for schoolwork, but especially to be in contact with children his own age.” Mother G.
	Quote 44	“I suppose what she missed most of all were her friends at school, and even though she was small, it's true that she still had very strong ties with certain children” Mother B
	**Recreating a familiar environment**	
	Quote 45	“It didn't change anything because I cooked what she wanted to eat. She didn't necessarily eat the hospital meal. I was the one who brought her in every day. I'd say to her, ‘well, what do you want tomorrow?’ and then I'd bring things back, I'd cook and then in Paris, you have access to everything. So, if she wanted a ‘colombo’, I'd make one. If she wanted a ‘fricassé’ of I don't know what, I'd make it. If she wanted red beans, I'd make them. There are grocery stores. Over there, it's the Pakistanis who keep all this exotic stuff, and then we have all the products we want.” Mother I
	Quote 46	“Creole is more than a language, it's a community. So not having anyone around who speaks Creole makes you feel a bit alone, a bit isolated, lost with your culture. […] but it's part of us, so we find ourselves speaking Creole even without knowing it, even without paying attention. You start a sentence in French and finish it in Creole.” Mother F
	Quote 47	“In his hospital room, yes, we brought back lots of photos of him. […] We'd brought back photos of us, we'd brought back his personal effects, his comforter. We tried to make it look… Yes, just like in his room at the hospital.” Mother H
	**Child's resilience**	
	Quote 48	“It's true that he made friends at [the reference center] […] It's true that at times he asked me when we were going to go home, that he wanted to see his grandma, his auntie, but I can't say that he's a child who suffered at all, frankly no. Fortunately.” Mother J
	Quote 49	“He got his friends back, he got his club back, he got his girlfriend back. He got his life back! He got his life back right away. His hair, so when we got home, his hair had already grown.” Father A
	**A timeless interlude**	
	Quote 50	“I., she didn't feel uprooted. But she's a special child, I'd say, who's hyper‐adaptable. And so she experienced it as, I'm going to say, almost an adventure. ‘I was sick, so it took up two pages in my life book. And then it's over, you move on to something else.” It's a parenthesis. And it's true that for all of us, it was a moment a little out of time, out of our habits, out of our usual culture in which we lived. But all that was in brackets. I think a lot of things were accepted because it was in the parenthesis. Mother I
	Quote 51	“It was pretty quick to get back into the routine and then almost pretend it never happened, in real life.” Mother F
	**Innocence of childhood**	
	Quote 52	“And the end was hard […] I mean, we were counting the days. We had to get home. Even if we wanted to settle down afterward, we had to leave the mainland France. It was too synonymous with the last few difficult months. And we had to go home, settle down, calm down, find our belongings, our habits, the warmth, our friends. We weren't home. We weren't home.” Mother I
	Quote 53	“She really wanted to go to school. […] And what she really missed was the presence of other children who weren't sick. That's what it reminds me of in terms of annoyances.” Mother D
	Quote 54	“I had bought 2‐3 puzzles. So that was good, we did puzzles sometimes. Drawings too. So frankly, I tried to keep him as busy as possible. […] He has good memories. Well, I mean, when I say he has good memories, it's really the games part, everything that's going on around him [apart from the care].”. Mother C
	Quote 55	“I know that his grandmother even sent him mangos from Martinique, so that was a real treat for him. He wanted them, he dreamed about them, he talked about them all the time. So, she made him a little parcel and sent it to him. And he tasted them, but I think he really liked them. On the plate, we gave him a lot of comfort, in any case.” Mother C
A positive feeling about the idea of partial care in Martinique	**Benefits of a comforting and familiar environment**	
	Quote 56	“It's complicated to have to leave, especially when you've just been told about something so serious. We'd rather stay surrounded by those closest to us, so that we have support.” Mother F
	Quote 57	“I think it's true that I would have been here [in Martinique], it's true that I would have had my mom. For the family circle, it would have been nice. [….] It's true that I would have had my family behind me, and that might have been more relaxing for me, because the pace I had over there wasn't really relaxing. In other words, really resting with, how should I say, a light sleep, in fact.” Mother C
	Quote 58	“It would be more advantageous already for my partner [mother E] because she has her two children, for my mother.” Father E
	Quote 59	“It's true that when you're alone, it's difficult. Oh yes, I think that's the worst thing, being too far away from the people you love, it's difficult. […] Maybe his granny could have come to see him, his parents, his friends. He could have seen more people.” Mother J
	Quote 60	“My mom, I think the closeness would have made it easier for us to keep in touch and have less of a feeling of being torn away, of having been torn away from home in fact.” Mother H
	**Reducing hardships**	
	Quote 61	“It would have had a colossal impact on her because we wouldn't have had to leave. We would have stayed surrounded by friends and family, so it wouldn't have changed our way of life. […] So, the treatment here would be more for the people around us, to stay surrounded more easily and not have to relearn how to live somewhere else, because you may be comfortable at [parent's house], but you're always at your best at home. So, it's a weight off our shoulders, and we don't have to ask ourselves the same questions.” Mother F
	Quote 62	“It's true, it would have been much better for me if G. had been cared for directly in Martinique. That's for sure.” Mother G
	Quote 63	“It's true that if you manage to set up a local unit, it will be all the better, because it will obviously be simpler for the parents. For the children too, because maybe when it comes to being able to get out of hospital for a while, the landmarks are there, the temperature is there, everything is pretty much the same.” Mother D
	Quote 64	“Because when we're there, we say to ourselves, especially at the parent's house, normally it's 2 months, we say to ourselves, we've got to find something. We've got to do, we've got to get a job to support ourselves. But at home, we've already got a job, we've already got a place to live, so we don't have that kind of questioning. […] Of course, it would only be positive. I can't think of a single negative thing about staying at home and being cared for at home.” Mother F
	**Limiting changes in daily lives**	
	Quote 65	“I really only see the fact that there's less of a change of scenery. And that may be trivial, but it's very important, the fact of eating something other than what we eat. Something else, but with different flavors. Because you can eat pasta, it's not a problem, we eat pasta everywhere, but we don't have the same spices, we don't have… That's it. So yes, I think that's what it's all about, being somewhere that's as close as we are, that resembles us, in fact.” Mother H
	Quote 66	“And again, I don't know, I can't say. Because it's true that in Martinique, we might have access to the river, the sea, but could she have had access to that? With her implantable chamber, I'm not sure. The same climatic and cultural conditions, but that's not why we leave. We leave for treatment. I really don't know. Frankly, I don't know. I don't know.” Mother I
	Quote 67	“He doesn't know, actually, and the others don't know what he might have looked like during treatment either. But yes, I think if we'd stayed, he could have gone back to school, seen his friends. They would have seen the change in his appearance. I think it would have been easier [to be in Martinique for treatment].” Mother H
	Quote 68	“It would have been much better [to be in Martinique for treatment], if only for schooling, because I could have, for example, brought homework, attended classes with another student, a mother of another child, and then he could have continued with his class. Everything is possible now. With the Internet, you can do so many things. I'm sure he could have continued his schooling at the hospital without any problems. Because he learns his lessons in bed, he does his exercises. When she [the teacher] gives the evaluation, she can send me a copy and I give it to G. and then I bring it back to him. There are a lot of things that are possible.” Mother G

### 3.1 The Role of the Parent: Focus on the Child and Management of Complex Logistics

#### 3.1.1 Hasty and Urgent Departure Induces Lack of Organisation

All of the parents interviewed reported a hasty and urgent departure. This led to a lack of organization. Symptomatology at the time of the child's diagnosis was not addressed in the questionnaire, but parents often needed to go back over the history leading up to the diagnosis, to explain the context of the departure (Quotes 1‐2‐3‐4).

#### 3.1.2 The Sick Child: A New Priority

Whatever concessions had to be made to focus on the child, the sick child imposed itself on the parents as a new priority to be put first (Quotes 5‐6).

#### 3.1.3 The Potomitan Parents

The parent felt it was his duty to cope with the ordeal, both mentally and physically. He wanted to reassure his child and support him in his care, despite his own worries. At the same time, his position as a parent was disturbed (Quotes 7‐8‐9).

#### 3.1.4 Lost in Translation

The complexity of parental organization lies in the long‐distance management of professional life, financial resources, two homes and siblings. In the FWI and in mainland France, the problems of work and housing were closely linked to financial problems, with considerable costs, especially for parents who rented accommodation and had no access to accommodation for the families of sick children (e.g., the Association of Families of Handicapped Children or the Ronald McDonald Foundation) (Quotes 10‐11‐12‐13).

#### 3.1.5 Struggling Through Adversity

In addition to employment and housing, these parents also had to manage other parental responsibilities such as the logistics of looking after siblings, as well as transport and winter clothing (Quotes 14‐15‐16).

### 3.2 The Interdependence of the Parent‐Child Pair With the Environment

#### 3.2.1 Children and Parents Standing Together

As a result of this experience, the parent‐child pair stood together and tried to preserve and protect each other within the family unit (Quotes 17‐18‐19‐20).

#### 3.2.2 A Shared Experience

It was a shared experience of difficulties, but sometimes a source of a positive balance (Quotes 21‐22‐23).

#### 3.2.3 Intricacies of Family Reorganization

The family unit suffered from this reorganization, with repercussions on siblings in particular, but also on the couple and on the parent as an individual (Quotes 24‐25‐26).

#### 3.2.4 The Other Side of the Coin

The parents seemed to experience more difficulties and anxiety than the children when they returned home after treatment, and some considered moving to mainland France (Quotes 27‐28‐29‐30).

#### 3.2.5 Family's Support Network

Parents mentioned a number of resources for themselves and their child: grandparents to look after siblings, but also to provide moral support for the child and the parent; associations with a strong presence in reference centers in mainland France or Martinique; sharing experiences with other parents; the parents' house; faith, sometimes described as an indispensable resource; and friends and family, both in mainland France and far away, as a source of reassurance (Quotes 31‐32‐33‐34‐35‐36).

#### 3.2.6 The Hospital: The Lighthouse in the Storm

In mainland France, the hospital is often an unfamiliar world for families. Nevertheless, it remains a trusted reference structure. The parents insisted on the importance of administrative support and guidance (Quotes 37‐38‐39).

### 3.3 Remaining a Child Above all Else: During Illness and in Long‐Distance Care

#### 3.3.1 Child's Acceptance of Care

Acceptance of the child's care was mixed, and the child's appearance, particularly the loss of hair, was seen as a difficulty (Quotes 40‐41).

#### 3.3.2 Child's Need for Support

The child's need to have their parents at their bedside was fundamental. The need for a circle of support, especially siblings, friends and classmates, was important to the sick child and contact was often maintained despite the distance (Quotes 42‐43‐44).

#### 3.3.3 Recreating a Familiar Environment

Families tried to recreate a familiar and reassuring environment for their child who had lost his or her daily routine and landmarks (Quotes 45‐46‐47).

#### 3.3.4 Child's Resilience

Most of the children were resilient and able to adapt to the ordeal and express their emotions. In particular, the children readapted quickly on their return home (Quotes 48‐49).

#### 3.3.5 A Timeless Interlude

For some children, the distance seemed to isolate this period of care in time and space, facilitating a return to pre‐diagnosis routines (Quotes 50‐51).

#### 3.3.6 Innocence of Childhood

The innocence of childhood seemed to have been preserved, according to parents' accounts of their child's preoccupations and sources of joy during the stay in mainland France, albeit with expressions of a lack and a certain nostalgia for home (Quotes 52‐53‐54‐55).

### 3.4 A Positive Feeling About the Idea of Partial Care in Martinique

#### 3.4.1 Benefits of a Comforting and Familiar Environment

There is an undeniable benefit for patients and their families in having at least some care provided locally or regionally. It seemed obvious to the parents that reducing the physical distance would facilitate the presence and emotional support of family and friends and thus limit the overall trauma to the family (Quotes 56‐57‐58‐59‐60).

#### 3.4.2 Reducing Hardships

Being in their original cultural environment would have allowed the children to maintain a semblance of normality by preserving their everyday landmarks without disrupting the family nucleus and its organization as much as possible (Quotes 61‐62‐63‐64).

#### 3.4.3 Limiting Changes in Daily Lives

Although care remains the main objective, having one's own environment is reassuring and comforting for both the child and the parents. In particular, school is an important reference point for the child's social life and daily routine (Quotes 65‐66‐67‐68).

## 4 Discussion

This qualitative study reports on the experiences of 10 families from FWI and FG treated for childhood cancer in mainland France. Parents expressed that the relocation of their child for treatment had an impact on the family unit, with the reorganization required having a negative impact on both their own and their child's experience at this difficult time.

Despite these challenges, parents maintained full confidence in the healthcare pathway and providers. They acknowledged that the implementation of complex organizational logistics was inevitable in order to support their child in the new forced living conditions. These families had to cope simultaneously with the diagnosis of a serious illness and the significant lifestyle changes required to support their sick child. This situation led to a sudden and complete change in their lifestyle, affecting several aspects, including financial stability, work commitments, relationships with siblings, material needs (such as housing and clothing), and dietary and cultural habits. Indeed, in the French overseas departments, the majority of the population is Afro‐Caribbean, and therefore these territories have a strong and specific history linked to the African territories, which has shaped their cultural heritage and can be very different from the cultural heritage of mainland France. These changes have taken place in the context of the overseas departments, where socio‐economic levels are generally lower than in mainland France, placing a double burden on these children and their families.

### 4.1 Clinical Implications

In pediatric oncology, parents are the child's most important resource. Effective care of a child with cancer must take into account the family context in order to support parents in fulfilling their primary role: caring for the sick child. Parents of children with cancer need to draw on internal and external resources to cope with the significant stress associated with their child's diagnosis and treatment [16]. For the parents interviewed, the abrupt reorganization caused by medical evacuation destabilized the family nucleus and made it more difficult to mobilize these resources.

A Swedish study of 395 parents of children with cancer reported a typical coping pattern: intense parental distress at diagnosis, followed by a return to normal functioning 3–6 months later [12]. This pattern was also observed among parents in this study, but they had the additional burden of managing complex family and financial logistics while coming to terms with the diagnosis of a serious illness. It is possible that this period of distress is prolonged due to the particular situation of the care pathway. Parents' emotional difficulties may affect their support, education and care of their sick child, as well as the experiences of siblings. There is a well‐established link between parental and child mental health according to scientific literature [11].

In addition, mothers noted differences in how mothers and fathers experienced these challenges, particularly in relation to the difficulties of returning home. Gender differences have been documented among parents of pediatric cancer patients, with fathers often focusing on the immediate problems at diagnosis and less on long‐term issues, in contrast to mothers. In this study, mothers were more likely to seek social connections to help stabilize their situation. [8, 11, 17, 18]. Both parents expressed a need for psychological support on return, a need that is echoed in the literature, particularly in the long‐term follow‐up of adolescents and young adults [19]. Even without the ordeal of uprooting, patients and their families may experience positive emotions such as relief and joy at the end of treatment, but they also face negative and ambivalent emotions, including a sense of uncertainty or heightened anxiety [20] like revealed in our study. Future research could explore whether these difficulties persist in the medium to long term by conducting follow‐up interviews with these parents.

Family‐centered psychosocial care is essential in pediatric oncology [16]. The transfer of a child from the FWI and FG to mainland France is often perceived as a form of expatriation. Apart from the language barrier, this experience is similar to that of a foreign child being cared for in France and requires systematic and in‐depth multidisciplinary reflection beforehand. The involvement of social services in both the home and host departments is crucial, as the parents interviewed pointed out. These families need more time than average for coordination and adaptation [5, 6].

Perceptions of greater family support, less conflict and easier parenting at diagnosis are associated with better marital adjustment [17]. Understandably, marital and family adjustment is more difficult in situations of geographical separation, as expressed in the parents interviewed, many of whom experienced significant distress during their hasty departure. A 2022 study of the concept of hope in parents of children with cancer found that hope was negatively correlated with parental psychological distress and adjustment dysfunction [21]. Consistent with the literature, some parents in this study found support in religion and felt reassured by medical care, particularly when the communication from healthcare providers was clear and realistic. Indeed, parents' hope, and consequently their resilience, can be strengthened by religiosity, spirituality and appropriate communication between parents and caregivers [21]. In Afro‐Caribbean populations, spirituality and religion may play a particularly important role as sources of support [22].

Distance care and sibling logistics represent complex adjustment challenges for families. Known risk factors for parental maladjustment include low socio‐economic status and unemployment [23]. According to the French National Institute for Statistics and Economic Studies, the population of the French overseas departments is already more socially vulnerable than that of mainland France, with higher unemployment rates and lower educational attainment, particularly in Martinique. Financial support was often perceived as insufficient to meet the needs of a two household, and the loss of a parent's job exacerbated financial instability. These difficulties were particularly acute for single‐parent families and those with several children. In 2024, the daily parental attendance allowance will be 64 euros per day, limited to 22 days per month, amounting to 1419 euros per month. Although the French social security system is notoriously more generous than its North American or British counterparts [24], this allowance may still be insufficient to cover all local and long‐distance costs for the family.

Parents also raised concerns about health insurance coverage, noting that only one parent is usually covered for pediatric medical evacuation (EVASAN), leaving the cost of a second parent or sibling as the responsibility of the family. This issue has been brought to the attention of policymakers and a recent decree has been published to cover the costs of a second carer for people from the French overseas departments [25].

The children in this study seemed to retain their childhood interests and adapted relatively well to being uprooted for cancer treatment. Their experiences overlapped with those of children isolated by hospitalization, as perceived through their parents' narratives [10, 26]. Despite the challenges, they showed a strong ability to reintegrate into their daily lives and to readjust quickly to their original environment. This contrasts with findings from other studies of cancer survivors, which often highlight more prolonged adjustment difficulties [14].

It is important to note, however, that the diagnoses of the children whose parents were interviewed were all recent (< 2 years) and, with the exception of one child, none had significant or disabling sequelae. While the children's behavior and attitudes did not appear to change dramatically during treatment away from home, some expressed a desire for the presence of their extended family and friends. Family cohesion, which was disrupted by distance during treatment, is known to be a key indicator of a child's quality of life [27]. According to the parents of our study, the children suffered from this separation but found sources of comfort in the play activities available in mainland France.

The potential benefits of providing at least some care closer to home could be significant. This would allow certain outpatient treatments to be carried out with less disruption to family routines and rhythms [28]. Parents often described their child's schooling as disjointed, although a social link with teachers and the original class was maintained, providing a degree of comfort for the child. Schooling is an important social pillar in a child's life, much like work is for adults, and the psychosocial impact of displacement can significantly affect this aspect.

Despite the period of remote care, the return to school was perceived as a return to normality and the children generally adapted well, except in one case where the physical consequences of the illness affected the reintegration. Moving, even temporarily, is not without consequences for a child, and recommendations have been developed to help prevent and resolve conflicts related to family moves. Isolation during prolonged hospitalization was explored in a qualitative study of 8 children treated for medulloblastoma. The findings showed that children who were isolated in hospital were removed from their usual environment and social experiences. Reducing this isolation should be a priority, and new information and communication technologies could help to bridge this gap by facilitating connections with their peers [26].

Although the psychological impact on children was not put forward in our study due to the focus on parents' perspective, some studies show that forcibly displaced children have higher rates of post‐traumatic stress disorder, depression and anxiety [9, 29]. Similar findings have been observed in the health context of displaced Canadian Aboriginal people who have been separated from their family roots and culture through forced medical resettlement [30].

Studying the factors that contribute to mental health problems in refugee children, as well as the mechanisms that support successful resettlement in host societies, enhances our understanding of the complex interplay between the adverse effects of displacement and the protective factors that these children inherently possess. For example, re‐establishing connections through valuing the culture of origin contributes to a sense of cultural continuity, which acts as a protective factor against suicide risk [31]. In our study, some parents described stronger bonds and closer family relationships after this ordeal, while others experienced a breakdown of the family unit and individual weakness impacting their children. Family cohesion can be improved by providing a partial care pathway closer to home, thereby increasing treatment adherence [7]. There was unanimous confidence in the care provided in mainland France. However, none of the parents interviewed had experienced treatment failure or palliative care situation.

Similar to our findings, siblings of patients, who were already struggling [13], often felt isolated and neglected, despite their parents' efforts. Those siblings who were able to spend time with their sick brother or sister during school holidays or return to school in mainland France reported better experiences. In addition, parents expressed guilt for leaving their other children behind in their place of origin [13].

This study supports the anticipated psychosocial benefits of shared care between reference centers and regional centers in the FWI and FG for pediatric oncology. Close collaboration with reference centers in the Paris region, together with shared management, would help to build confidence when patients and families return home from the FWI and FG. This approach would facilitate the delivery of a greater proportion of care as close as possible to where families live, especially given the significant geographical distance of the FWI and FG. Such a strategy is in line with French legislation, which aims to provide care for serious illnesses as close to home as possible. However, the development of regional centers needs to be carefully coordinated with the reference centers and to be prudent and strategic. However, the development of regional centers needs to be carefully coordinated with the reference centers and be prudent and strategic. It is clear that these benefits are more likely to be expected by families with regional ties and a comfortable capacity for medium‐term stays provided by family ties.

These regional centers would ensure the presence of well‐trained medical and paramedical staff with a thorough knowledge of the relevant pathologies, allowing the safe management of diagnostic emergencies, complications and treatment‐related toxicities.

In addition, just as home hospitalization has been shown to reduce children's anxiety and fear of treatment by providing care in a familiar and safe environment, regional therapeutic care would allow families to rely on their local support networks. This would limit the psychological and financial burden associated with long‐distance relocation in a context of vulnerability related to the severity of illness and uncertainty of recovery [32].

This study has several strengths: it is the first qualitative study conducted among this population of overseas parents whose children have been treated for childhood cancer through a unique healthcare pathway. The study was well received by the families and provided a valuable platform for parents, many of whom expressed satisfaction in sharing their experiences. For some, it provided a sense of closure as the geographical distance allowed them to compartmentalize these months of care. The unique nature of the healthcare pathway, which lends itself well to Interpretative Phenomenological Analysis, enabled these narratives to reveal non‐somatic issues faced by families, which can be considered additional adverse effects of childhood cancer treatment.

### 4.2 Study Limitations

However, this study has some limitations. The inclusion criteria excluded parents whose children were receiving palliative care, resulting in a recruitment bias toward diseases with a more favorable prognosis. It is important to note that palliative care is often provided closer to home, which may influence parents' perceptions differently. In addition, the interviews took place sometime after the child's treatment in mainland France, introducing the possibility of recall bias, although the diagnoses were all less than 5 years old.

In our study, the child's experience was conveyed through the parents' perspective. Future research should consider interviewing the sick children directly, as well as their siblings and co‐parents who may have remained in the Overseas Territory. Another limitation is that the majority of patients were from the FWI (> 80%), which may bias the results in terms of cultural realities, as the cultural beliefs and practices of the FWI and FG populations are not entirely similar.

A quantitative study of the socio‐demographic parameters of EVASAN in the FWI and FG could complement these results, in line with a similar study carried out on the island of Reunion [4]. In addition, a qualitative study focusing on parents of children with chronic illnesses other than cancer requiring prolonged or repeated treatment in mainland France would be valuable. Such a study could shed light on the unique challenges of managing chronic illness and help to distinguish the experience from the heightened fear of death that accompanies a childhood cancer diagnosis.

## 5 Conclusion

This qualitative study, the first of its kind, explores the experiences of families from the FWI and FG whose children are being treated for childhood cancer in mainland France. Relocation for treatment has a significant impact on family dynamics, requiring a complex reorganization that often disrupts the family nucleus. Parents express difficulties in mobilizing resources to cope with the stress of diagnosis and treatment, while maintaining a high level of trust in the care system and health professionals. They emphasize the need for psychological support, especially when they return home, and often find comfort in religion and communication with caregivers. The financial and social challenges are exacerbated by the high rates of poverty and unemployment in the overseas departments, which particularly affect single‐parent and large families. Despite their adaptability, children suffer from separation from their families and siblings feel isolated and neglected. Part‐time care close to home could alleviate these challenges, allow families to draw on their support networks and reduce psychological and financial costs. The findings call for greater involvement of social services and in‐depth multidisciplinary thinking to better support these families. Further studies are needed to expand on these findings and propose solutions tailored to the specific challenges faced by families in the French overseas departments. In conclusion, we believe that partial care closer to home would strengthen family cohesion, improve compliance with medical treatments and limit the psychological and financial costs of adapting to a new life imposed by a child's serious illness.

## Author Contributions

Conceptualization, C.V., A.O., and A.F.: data verification and curation, C.V., A.O., and A.F.: investigation, C.V., A.O., and A.F.: formal analysis, C.V., A.O., and A.F.: methodology, C.V., A.O, and A.F.: writing – original draft, C.V., A.O., and A.F.: writing – review and editing, all authors.

## Ethics Statement

All procedures performed in this study were in accordance with the ethical standards of the institutional and/or national research committee and with the 1964 Helsinki declaration and its later amendments or comparable ethical standards. The study was approved by the Institutional Review Board of the Martinique university hospital under number 2022/227.

## Consent

All participants provided written informed consent for participation and publication.

## Conflicts of Interest

The authors declare that the research was conducted in the absence of any commercial or financial relationships that could be construed as a potential conflict of interest.

## Supporting information

Supporting Information S1

## Data Availability

The data that support the findings of this study will be available upon reasonable request to the corresponding author of the study.

## References

[1] B. P. Kurtz and A. N. Abrams , “Psychiatric Aspects of Pediatric Cancer,” Child and Adolescent Psychiatric Clinics of North America 19, no. 2 (2010): 401–421: x‐xi, 10.1016/j.chc.2010.01.009. 20478507

[2] M. L. Stuber , K. A. Meeske , K. R. Krull , et al. “Prevalence and Predictors of Posttraumatic Stress Disorder in Adult Survivors of Childhood Cancer,” Pediatrics 125, no. 5 (May 2010): e1124‐34–e1134, 10.1542/peds.2009-2308. 20435702 PMC3098501

[3] A. Felix , F. Delion , B. Suzon , et al. “Systemic Lupus of Pediatric Onset in Afro‐Caribbean Children: A Cohort Study in the French West Indies and French Guiana,” Pediatric Rheumatology 20, no. 1 (2022): 95, 10.1186/s12969-022-00759-7, November 12, 2022. 36371201 PMC9652926

[4] M. Drean , D. Orbach , E. Chirpaz , et al. “Pediatric Medical Evacuations From Reunion Island to Metropolitan France,” Bulletin du cancer 110, no. 2 (2023): 174–183, 10.1016/j.bulcan.2022.11.010. 36503611

[5] N. Augier , F. Grialou , and G. Leverger , “Social Management of Children Coming From Foreign Countries: Experience in a Specialized Center,” Archives of Pediatrics 12, no. 6 (2005): 856–857, 10.1016/j.arcped.2005.04.008. 15904828

[6] J. Gaudichon , S. Toscani , S. Cohen‐Gogo , et al. “Care Management for Foreign Children, Adolescents, Young Adults With Cancer, and Their Families,” Pediatric Blood and Cancer 64, no. 6 (juin 2017), 10.1002/pbc.26336. 27905679

[7] B. H. Fiese and R. S. Everhart , “Medical Adherence and Childhood Chronic Illness: Family Daily Management Skills and Emotional Climate as Emerging Contributors,” Current Opinion in Pediatrics 18, no. 5 (October 2006): 551–557, 10.1097/01.mop.0000245357.68207.9b. 16969171

[8] A. L. H. Pai , R. N. Greenley , A. Lewandowski , D. Drotar , E. Youngstrom , and C. C. Peterson , “A Meta‐Analytic Review of the Influence of Pediatric Cancer on Parent and Family Functioning,” Journal of Family Psychology 21, no. 3 (2007): 407–415, 10.1037/0893-3200.21.3.407. 17874926

[9] C. R. Cayabyab , P. O’Reilly , A. M. Murphy , and C. O’Gorman , “Psychological Morbidity Among Forcibly Displaced Children‐A Literature Review,” Irish Journal of Medical Science 189, no. 3 (2020): 991–997, 10.1007/s11845-020-02186-7. 31993955

[10] E. N. Alvarez , M. C. Pike , and H. Godwin , “Children’s and Parents’ Views on Hospital Contact Isolation: A Qualitative Study to Highlight Children’s Perspectives,” Clinical Child Psychology and Psychiatry 25, no. 2 (2020): 401–418, 10.1177/1359104519838016. 30990077

[11] L. M. Dahlquist , D. I. Czyzewski , and C. L. Jones , “Parents of Children With Cancer: A Longitudinal Study of Emotional Distress, Coping Style, and Marital Adjustment Two and Twenty Months After Diagnosis,” Journal of Pediatric Psychology 21, no. 4 (1996): 541–554, 10.1093/jpepsy/21.4.541. 8863463

[12] A. L. Norberg , F. Lindblad , and K. K. Boman , “Coping Strategies in Parents of Children With Cancer,” Social Science & Medicine 60, no. 5 (2005): 965–975, 10.1016/j.socscimed.2004.06.030. 15589667

[13] C. A. Gerhardt , V. Lehmann , K. A. Long , and M. A. Alderfer , “Supporting Siblings as a Standard of Care in Pediatric Oncology,” Pediatric Blood and Cancer 62, no. 5 (2015): S750‐804–S804, 10.1002/pbc.25821. 26700924

[14] K. Darabos and J. S. Ford , “Basically, You Had Cancer and now You Don’t: Exploring the Meaning of Being a Cancer Survivor Among Adolescents and Young Adult Cancer Survivors,” Journal of Adolescent and Young Adult Oncology 9, no. 4 (août 2020): 534–539, 10.1089/jayao.2019.0176. 32239966 PMC7640746

[15] Smith, J.A. and Osborn, M. (2008) “Interpretative Phenemenological Analysis.” In Smith, J.A. , Ed., Qualitative Psychology A Practical Guide to Research Methods, Sage, 53‐80. ‐ References ‐ Scientific Research Publishing [Internet]. [cité 11 juill 2024]. Disponible sur, https://www.scirp.org/reference/referencespapers?referenceid=1472730.

[16] J. A. Kearney , C. G. Salley , and A. C. Muriel , “Standards of Psychosocial Care for Parents of Children With Cancer. Pediatr Blood Cancer,” 62, no. 5 (2015): S632–S683, 10.1002/pbc.25761. PMC5066591 26700921

[17] W. Burns , K. Péloquin , S. Sultan , et al. “A 2‐year Dyadic Longitudinal Study of Mothers’ and Fathers’ Marital Adjustment When Caring for a Child With Cancer,” Psycho‐Oncology 26, no. 10 (October 2017): 1660–1666, 10.1002/pon.4189. 27278682

[18] J. E. Hoekstra‐Weebers , J. P. Jaspers , W. A. Kamps , and E. C. Klip , “Gender Differences in Psychological Adaptation and Coping in Parents of Pediatric Cancer Patients,” Psycho‐Oncology 7, no. 1 (1998): 26–36, 10.1002/(sici)1099-1611(199801/02)7:1<26::aid-pon315>3.0.co;2-0. 9516648

[19] V. Baudry , M. Girodet , M. Lochmann , et al. “Supportive Care Needs of Adolescents and Young Adults 5 Years After Cancer: A Qualitative Study,” Frontiers in Psychology 15 (2024): 1268113, 10.3389/fpsyg.2024.1268113. 38746913 PMC11091414

[20] M. Conway Keller , C. King , L. Hart , et al. “The End of Cancer Treatment Experience for Children, Adolescents, and Their Parents: A Systematic Review of the Literature,” Journal of Psychosocial Oncology 38, no. 5 (2020): 573–591, 10.1080/07347332.2020.1769795. 32602790

[21] I. J. Eche , I. M. Eche , C. Pires , C. Isibor , A. Achibiri , and T. Aronowitz , “A Systematic Mixed‐Studies Review of Hope Experiences in Parents of Children With Cancer,” Cancer Nursing 45, no. 1 (2022): E43‐58–E58, 10.1097/ncc.0000000000000841. 32657902

[22] L. M. Chatters , R. J. Taylor , K. M. Bullard , and J. S. Jackson , “Race and Ethnic Differences in Religious Involvement: African Americans, Caribbean Blacks and Non‐Hispanic Whites,” Ethnic and Racial Studies 32, no. 7 (2009): 1143–1163, 10.1080/01419870802334531. 20975850 PMC2962581

[23] M. J. Dunn , E. M. Rodriguez , A. S. Barnwell , et al. “Posttraumatic Stress Symptoms in Parents of Children With Cancer Within Six Months of Diagnosis,” Health psychology 31, no. 2 (2012): 176–185, 10.1037/a0025545. 21942750 PMC4243458

[24] K. Chevreul , K. Berg Brigham , I. Durand‐Zaleski , and C. Hernandez‐Quevedo , “France: Health System Review,” Health Syst Transit 17, no. 3 (2015): 1–218: xvii. 26766545

[25] I. I. I. Chapitre , “La Continuité Territoriale Entre Les Collectivités d’outre‐mer Et Le Territoire Métropolitain (Articles L1803‐1 À L1803‐18) ‐ Légifrance [Internet],”: [cité 8 avr 2025]. Disponible sur, https://www.legifrance.gouv.fr/codes/section_lc/LEGITEXT000023086525/LEGISCTA000023070046/.

[26] J. L. Sawyer , F. Mishna , E. Bouffet , M. Saini , and R. Zlotnik‐Shaul , “Bridging the Gap: Exploring the Impact of Hospital Isolation on Peer Relationships Among Children and Adolescents With a Malignant Brain Tumor,” Child and Adolescent Social Work Journal 40, no. 1 (2023): 91–105, 10.1007/s10560-021-00764-x. 34025015 PMC8130807

[27] S. Santos , C. Crespo , M. C. Canavarro , and A. E. Kazak , “Family Rituals and Quality of Life in Children With Cancer and Their Parents: The Role of Family Cohesion and Hope,” J Pediatr Psychol. août 40, no. 7 (2015): 664–671, 10.1093/jpepsy/jsv013. PMC4542831 25775914

[28] B. Stevens , R. Croxford , P. McKeever , et al. “Hospital and Home Chemotherapy for Children With Leukemia: A Randomized Cross‐Over Study. Pediatr Blood Cancer,” 47, no. 3 (2006): 285–292, 10.1002/pbc.20598. 16200556

[29] A. Javanbakht and L. R. Grasser , “Biological Psychiatry in Displaced Populations: What We Know, and What We Need to Begin to Learn,” Biological Psychiatry: Cognitive Neuroscience and Neuroimaging 7, no. 12 (2022): 1242–1250, 10.1016/j.bpsc.2022.05.001. 35580738 PMC9678009

[30] E. Fuller‐Thomson , S. Lee , R. E. Cameron , P. Baiden , S. Agbeyaka , and T. M. Karamally , “Aboriginal Peoples in Complete Mental Health: A Nationally‐Representative Canadian Portrait of Resilience and Flourishing,” Transcult Psychiatry 57, no. 2 (2020): 250–262, 10.1177/1363461519885702. 31747867

[31] V. Deriu , L. Benoit , M. R. Moro , and J. Lachal , “Suicidal Thoughts and Suicide Attempts in Adolescence Among Migrants,” Soins ‐ Psychiatrie 39, no. 316 (2018): 22–26, 10.1016/j.spsy.2018.03.005. 29753434

[32] L. Ranney , M. C. Hooke , and K. Robbins , “Letting Kids be Kids: A Quality Improvement Project to Deliver Supportive Care at Home After High‐Dose Methotrexate in Pediatric Patients With Acute Lymphoblastic Leukemia,” Journal of Pediatric Hematology/Oncology Nursing 37, no. 3 (2020): 212–220, 10.1177/1043454220907549. PMC7492745 32102635

