# Supplementary material for: Study of Uprooting in Pediatric Cancer Care for Children From the French West Indies and Guiana Treated in Mainland France: A Qualitative Study
Source: Psychooncology. 2025 Jul 29;34(8):e70242. doi: 10.1002/pon.70242 (PMC12306847; doi:10.1002/pon.70242)
Supplement: Supplementary file 1 — Supporting Information S1 [file PON-34-e70242-s001.docx]

# Supplementary data

## Supplementary table 1: Questionnaire / Interview guide

Dissertation title: **Study of the subjective experience of uprooting by the main caregiver of children cared for in mainland France following a cancer diagnosis in the West Indies**.

General socio-demographic data:

*Known as the “icebreaker” questions, these first questions are designed to provide general information about the child and parent, as well as to provide information for data anonymization.*

1. What is the **first name** of your child who has had cancer? How **old** was he or she at the time of **diagnosis,** and how old is he or she **today?** What **type of cancer** did your child have? How **long** did you spend in metropolitan France for treatment?

2. Could you describe your **family situation at the time of diagnosis?**

*Follow-up questions:* where did *you live? with whom? what grade was your child in? what was your profession and that of your spouse at that time in your life*.

3. What was your and your child's **connection with metropolitan France?**

In mainland France:

*The aim is to find out about the children's psychological, social (especially family) and cultural conditions and experiences, as described by their parents, in relation to the necessary move to mainland France.*

4. Could you tell me about **the organization of your departure?**

*Follow-up questions: how did you organize your belongings/clothes?* *What about your income, your accommodation, your means of mobility on the spot?*

5. How did you and your child **settle in** France?

*Follow-up questions: with regard to immersion in* ***metropolitan culture?*** *in terms of language/use of Creole? in terms of religion? eating habits?*

*How did you settle in? How did you decorate his room?*

6. Could you tell me about **your child's behavior** at the time of departure (announcement)? Then during the stay in France?

*Follow-up question: Vis-à-vis yourself?* *Towards others?* *How did your child integrate with the other children on site?* *How did he cope with other children's illnesses, and in particular with any deaths?*

7. Could you describe your child's **overall mood** during this period?

*Follow-up questions: How was his sleep? his mood? his concerns?*

8. How do you think he felt about the **separation from his usual environment**?

Follow-up question: separation from friends? family? school environment? extra-curricular activities? romantic relationships?

9. How did his **schooling** go during his stay in metropolitan France?

Family relationships:

*The aim is to find out about the experiences of the sick child's siblings and the quality of long-distance family relationships.*

10. Could you tell me about your **other children**, if you have any? Would it be possible to tell me how you think they experienced this period?

11. Could you tell us about the **relationship between yourself and other members of your family** at a distance?

12. How were **your child's relationships with other** long-distance family members? And with his friends?

Feedback:

*The aim is to assess how difficult it was for your child to return home.*

13. On your return, how has your child **re-adapted**?

*Follow-up question: schooling, place in siblings, relationship with the metropolis, sleep, friendships, etc.*

14. When you returned, how did you **re-adapt**?

*Follow-up questions: with regard to the repercussions on your professional activity, your income, your housing? and in your relationship?*

*Has a permanent move to mainland France been considered?*

Prospects for improvement:

*In order to improve care for these children and their families, and to better support them in this uprooting process, it is essential to ask their opinion on what they feel could be put in place.*

15. With hindsight, what do you think could be **done** **to improve the care experience** of West Indian and Guyanese children with cancer, and that of their families?

How do you think treatment in Martinique would have affected the experience you've just described?
